# Structural and Catalytic Differences between Two FADH_2_-Dependent Monooxygenases: 2,4,5-TCP 4-Monooxygenase (TftD) from *Burkholderia cepacia* AC1100 and 2,4,6-TCP 4-Monooxygenase (TcpA) from *Cupriavidus necator* JMP134

**DOI:** 10.3390/ijms13089769

**Published:** 2012-08-06

**Authors:** Robert P. Hayes, Brian N. Webb, Arun Kumar Subramanian, Mark Nissen, Andrew Popchock, Luying Xun, ChulHee Kang

**Affiliations:** 1 Department of Chemistry, Washington State University, Pullman, WA 99164, USA; E-Mails: robert_hayes@wsu.edu (R.P.H.); brian.webb1@email.wsu.edu (B.N.W.); subramanianarunk@gmail.com (A.K.S.); mnissen@wsu.edu (M.N.); 2 School of Molecular Biosciences, Washington State University, Pullman, WA 99164, USA; E-Mail: popchoa@wsu.edu

**Keywords:** TCP, flavin, bioremediation, monooxygenase, TcpA, TftD, crystal structure

## Abstract

2,4,5-TCP 4-monooxygenase (TftD) and 2,4,6-TCP 4-monooxygenase (TcpA) have been discovered in the biodegradation of 2,4,5-trichlorophenol (2,4,5-TCP) and 2,4,6-trichlorophenol (2,4,6-TCP). TcpA and TftD belong to the reduced flavin adenine dinucleotide (FADH_2_)-dependent monooxygenases and both use 2,4,6-TCP as a substrate; however, the two enzymes produce different end products. TftD catalyzes a typical monooxygenase reaction, while TcpA catalyzes a typical monooxygenase reaction followed by a hydrolytic dechlorination. We have previously reported the 3D structure of TftD and confirmed the catalytic residue, His289. Here we have determined the crystal structure of TcpA and investigated the apparent differences in specificity and catalysis between these two closely related monooxygenases through structural comparison. Our computational docking results suggest that Ala293 in TcpA (Ile292 in TftD) is possibly responsible for the differences in substrate specificity between the two monooxygenases. We have also identified that Arg101 in TcpA could provide inductive effects/charge stabilization during hydrolytic dechlorination. The collective information provides a fundamental understanding of the catalytic reaction mechanism and the parameters for substrate specificity. The information may provide guidance for designing bioremediation strategies for polychlorophenols, a major group of environmental pollutants.

## 1. Introduction

Polychlorophenols, such as 2,4,5- and 2,4,6-trichlrophenol (TCP) are recalcitrant pollutants introduced into the environment through their popular use as preservatives in wood industry, herbicides in agriculture, and as general biocides in consumer products [1,2]. Their persistence in the environment can be attributed to halide substitution rendering them resistant to microbial degradation. Due to desired substitutions and the lack of specificity in some of the manufacturing processes, different kinds of polychlorophenol isomers were produced. In addition, various forms of chlorophenols have been released as metabolic intermediates during the biodegradation of the mother compounds. For example, 2,4,5-TCP is the first metabolic intermediate in the degradation of 2,4,5-trichlorophenoxyacetate, a major ingredient of “Agent Orange” used in Vietnam for defoliation [3]. 2,4,6-TCP is also produced during the natural degradation process of a fungicide, prochloraz [4]. Human exposure to those polychlorophenols can lead to acute hyperthermia, convulsions, and rapid death, with long-term health effects resulting from mutagenicity and carcinogenicity [5]. Further health concerns exist with carcinogenic chlorinated dibenzo-*p*-dioxins and dibenzofurans [6], which are produced from polychlorophenols either during manufacturing processes [5] or by means of biotransformation in soils [7]. Major toxicity of chlorophenols is due to the uncoupling of mitochondrial oxidative phosphorylation and subsequent convulsions. Acute toxicity from industrial exposure has been demonstrated within 20 minutes of a worker being accidentally splashed on the arm and leg with 2,4-dichlorophenol leading to convulsions, collapse and death shortly thereafter [8]. Runoff from pesticide degradation, contaminated food supply and the chlorination of drinking water and waste water are the environmental sources of human exposure to chlorophenols. The Environmental Protection Agency has determined the presence of chlorophenols in at least 116 of the 1,467 National Priority List (NPL) serious hazardous waste sites targeted for long-term federal cleanup activities. However, the total number of NPL sites evaluated for chlorophenols has not been established and thus may increase as more sites are evaluated [9].

**Figure 1:**
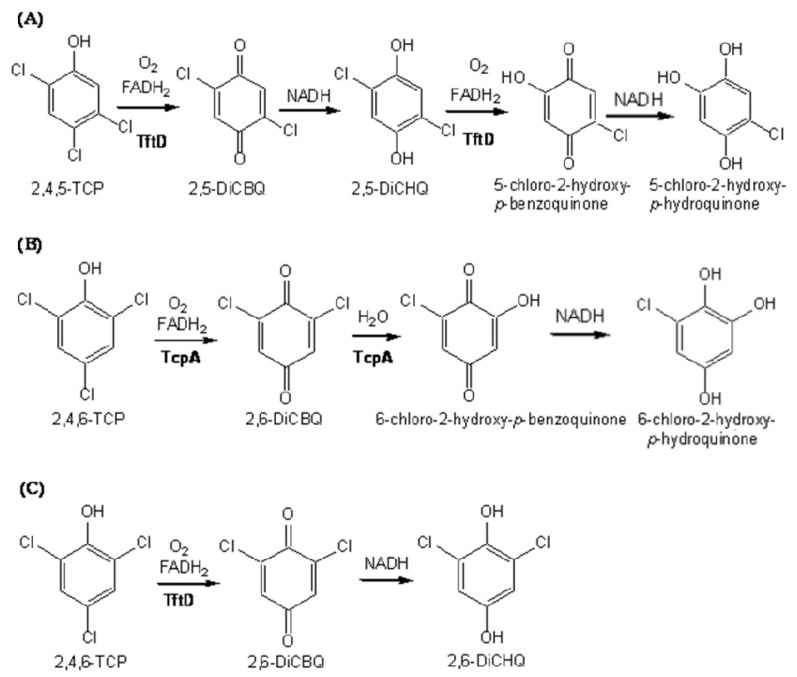
(**a**) 2,4,5-TCP 4-monooxygenase (TftD) oxidizes 2,4,5-TCP to 2,5DiCBQ which is reduced to 2,5-DiCHQ. Then, TftD oxidizes the latter to 5-chloro-2-hydroxy-*p*-benzoquinone, which can be nonenzymatically reduced to 5-chloro-2-hydroxy-*p*-hydroquinone; (**b**) 2,4,6-TCP 4-monooxygenase (TcpA) oxidizes 2,4,6-TCP to 2,6-DiCBQ, which is immediately converted to 6-chloro-2-hydroxy-*p*-benzoquinone by hydrolytic dechorination and reduced to 2,6-DiCHQ (**c**) TftD oxidizes 2,4,6-TCP to 2,6-DiCBQ, which is chemically reduced to 2,6-DiCHQ.

Recently, two microorganisms that degrade TCPs have been characterized. *Burkholderia cepacia* AC1100 can mineralize 2,4,5-TCP [10,11], and *Cupriavidus necator* JMP134 can completely degrade 2,4,6-TCP [12]*,* providing model systems for the study of their biodegradation. *C. necator* JMP134 degrades 2,4,6-TCP to 6-chlorohydroxyquinol (6-CHQol) before ring-cleavage [13]; whereas *B. cepacia* AC1100 converts 2,4,5-TCP to 5-chlorohydroxyquinol (5-CHQol) before ring cleavage [14]. Dechlorination of polychlorinated aromatic compounds is critical in their biodegradation because partial or complete dechlorination often must occur before ring-cleavage dioxygenases can open aromatic rings [15]. From our research and that of others, it is clear that the first step in polychlorophenol degradation is oxidation at the *para*-position (opposite to the phenolic group) catalyzed by a monooxygenase. If the *para-*position is chlorinated, the direct product is a quinone (Figure 1), which can then be reduced to the corresponding quinol by NADH either chemically [16] or by a quinone reductase [17,18].

The first enzyme in the 2,4,5-TCP degradation pathway of *B. cepacia* AC1100, 2,4,5-TCP 4-monooxygenase (TftD), is responsible for the conversion of 2,4,5-TCP to 5-chloro-2-hydroxy-*p*-hydroquinone (5-CHQ). The first enzyme in the 2,4,6-TCP degradation pathway of *C. necator* JMP134, 2,4,6-TCP 4-monooxygenase (TcpA), is responsible for the conversion of 2,4,6-TCP to 6-chloro-2-hydroxy-*p*-hydroquinone (6-CHQ). TftD and TcpA, share 65% sequence identity with no gaps in their sequence alignment. However, the substrate and resulting product of each enzyme show noticeable difference. For example, TftD and TcpA both use 2,4,6-TCP as a substrate; however, the two enzymes produce different end products (Figure 1B,C). TftD oxidizes 2,4,6-TCP to only 2,6-dichloro-*p*-benzoquinone (2,6-DiCBQ), which is released and then reduced to 2,6-dichloro-*p*-hydroquinone (2,6-DiCHQ). TcpA converts 2,4,6-TCP to 2,6-DiCBQ by oxidative reaction, followed by removing another chlorine by hydrolytic reaction, producing 6-chloro-2-hydroxy-*p*-hydroquinone [13] (Figure 1B). Therefore, TcpA catalyzes the sequential dechlorination without releasing the intermediate, 2,6-DiCBQ. Neither TcpA nor TftD use 2,6-DiCHQ as a substrate.

Both TcpA and TftD belong to the newly discovered reduced flavin adenine dinucleotide (FADH_2_)-dependent monooxygenases, which uniquely use FADH_2_ as a cosubstrate rather than a cofactor [19]. Several FADH_2_-dependent monooxygenases, such as TcpA and TftD, have been discovered in the biodegradation pathways of various aromatic compounds [20–23]. In the catalytic degradation of chlorophenols, the formation of a flavin-peroxide intermediate has been proposed, thus protection or stabilization of the FAD-peroxide intermediate from rapid hydrolysis seems crucial for flavin-dependent monooxygenases [24–28]. These monooxygenases uniquely have a small component partner protein (NADH:FAD oxidoreductase) with a flavin molecule either as a tightly bound substrate or as a prosthetic group from which reduced flavins are provided [21,29–31]. They are TftC and TcpX for *B. cepacia* and *C. necator* respectively. These enzyme pairs are also referred to as the two-component flavin-diffusible monooxygenase (TC-FDM) family.

This work utilized a crystallographic approach coupled with computational substrate docking to investigate the differences in catalytic mechanism between TcpA and TftD. A detailed understanding of the catabolic pathway for a specific polychlorophenol molecule, including substrate specificity and the catalytic mechanism of the participating enzymes, is essential for designing an effective bioremediation process for TCPs.

## 2. Results and Discussion

### 2.1. Overall Structure of TcpA

Recombinant TcpA from C. *necator* JMP134 was crystallized in the space group C2, with cell dimensions of a = 107.07 Å, b = 180.05 Å, c = 110.20 Å, β = 97.87 and there were four TcpA molecules in the asymmetric unit. A summary of the crystallographic data of 2.0 Å resolution is given in Table 1.

**Figure 2:**
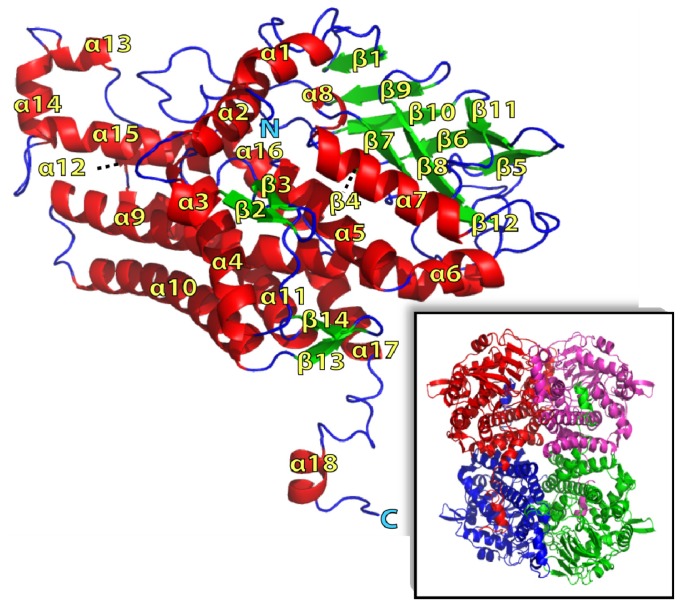
Ribbon diagram representing the crystal structure of the TcpA subunit. Secondary structure elements have been numbered sequentially as α1-α18 and β1-β14. *N* and *C* refer to the N- and C-terminal regions, respectively. α–helices were shown in red and β-strands were shown in green. Arrangement among the four subunits of TcpA was presented as an inset.

The individual TcpA molecules consisted of fourteen β-strands and eighteen α-helices (Figure 2) and could be divided into three sequential segments based on their constituent secondary structural elements. However, these three segments were interconnected in the 3D structure without providing any separate domain. The first segment from residues 1 to 146 was composed of seven α-helices (α1-α7) and three very short β-strands (β1-β3). The second segment, which was composed of residues 147–273, has nine β-strands (β4-β12) and one α-helix (α8). The third *C*-terminal segment was composed of ten α-helices (α9-α18) and two short β-strands (β13 and β14). Noticeably, all of the ten helices in the C-terminal segment, α9-α18, were involved in an inter-subunit interaction. All the nine β-strands in the second segment, β4-β12, were arranged in such a way to appear almost like a β-barrel. At one side of this barrel, the β1 strand from the first segment formed a continuous β-sheet with β-strands from the second segment in the order of β1-β5-β6-β9-β10-β11. The remaining four strands in the segment covered the other side of the same barrel in the order of β4-β7-β8-β12 (Figure 2). The amino acid side chains of those ten β-strands and the connecting loops between them established a hydrophobic core inside of the barrel.

Another noticeable structural feature was that three long α-helices, α10, α11 and α12, and three short helices, α15, α16 and α17, which were all from the C-terminal segment, formed a sizeable helix bundle together with two short α-helices from the first segment, α4 and α5. The surfaces of most of those α-helices were hydrophobic establishing a hydrophobic interaction among them.

The corresponding electron densities for the C-terminal residues 484–515 were not visible, probably due to being disordered. In addition, the temperature factors for residues 156–170, an exposed loop connecting α4 and α5, showed higher temperature factors than the rest of the molecule and were not visible likely due to disorder.

**Figure 3:**
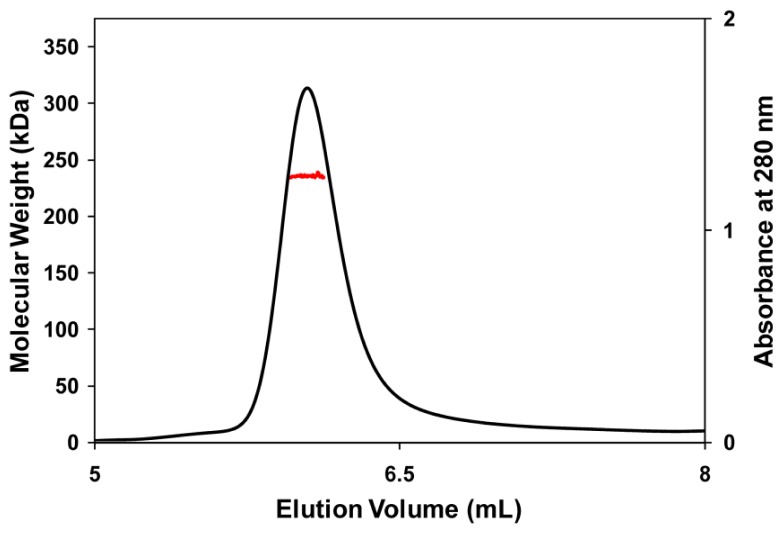
Elution profile of TcpA with multi-angle laser light-scattering (MALLS). Elution profile was shown as absorbance *versus* elution time with a thin black line representing changes in absorbance at 280 nm. The red line indicated the molecular mass calculated from the light scattering ultimately illustrating the tetrameric nature of TcpA.

Applying a two-fold symmetry operation to one of the two dimers in the asymmetric unit generates one tightly associated tetramer in a non-crystallographic *D_2_* symmetric assembly (Figure 2 inset). The tetrameric nature of TcpA in solution was confirmed by the elution profile and molecular weight estimates from a multi-angle laser light scattering (MALLS) experiment (Figure 3). The observed tetramer interface had an extensive network of inter-subunit interaction among the C-terminal α-helices. In particular, α17 and α18 extended out of the individual subunit to embed itself into the hydrophobic surface made by α9, α10 and α15 of the adjacent subunit. In addition, a large portion of two long and anti-parallel C-terminal helices, α10 and α11, from two neighboring subunits contacted from each other in a perpendicular orientation generating a substantial hydrophobic interface between subunits. These hydrophobic interfaces involved residues such as Val327, Leu361, Phe365, Leu451, Ile454 and Met459. Three α-helices at the C-terminal segment also established another type of subunit interaction together with the above-mentioned β-barrel like motif. That is, α13, α14, α16, their connecting loops, and the surface exposed residues in one side of the barrel formed tightly associated non-crystallographic 2-fold related interface. This interface possessed tightly packed hydrophobic residues; Val188, Val258, Leu390, Trp406 and Phe407. However, contrary to the above-mentioned hydrophobic interface involving α17 and α18, a substantial amount of polar inter-subunit interaction was also observed in this interface formed by the β-barrel and three helices, which included Asp160, Glu234, Lys238, Asp251, Asp260, Arg387, Gln397, Glu401, Asn412, Arg421, Arg429 and Asp435.

### 2.2. FADH_2_ Binding Site

**Figure 4:**
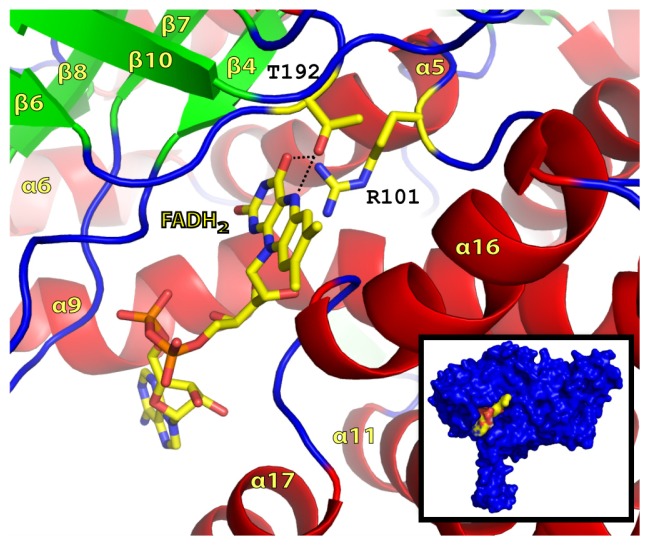
Flavin Adenine Dinucleotide binding site in TcpA. Autodock positioning of FAD in the active site of TcpA. Molecular surfacing illustrated Van der Waals interactions of FAD surface with active site surface (Inset).

In the apo-structure of TcpA, a unique entry site and binding pocket for FADH_2_ were clearly distinguishable (Figure 4). The perimeter of the widely open entry site was made with three flexible loops: (i) between β4 and β5; (ii) between β7 and β8; and (iii) between α16 and α17 (Figure 4). Among those three, the first and third loops had high temperature factors and showed the most heterogeneous conformations among the four subunits, reflecting their high level of flexibility. The residues lining this entry site were predominantly hydrophilic and a few ordered solvent molecules were located in the entry site of the determined apo-form structure of TcpA.

**Figure 5:**
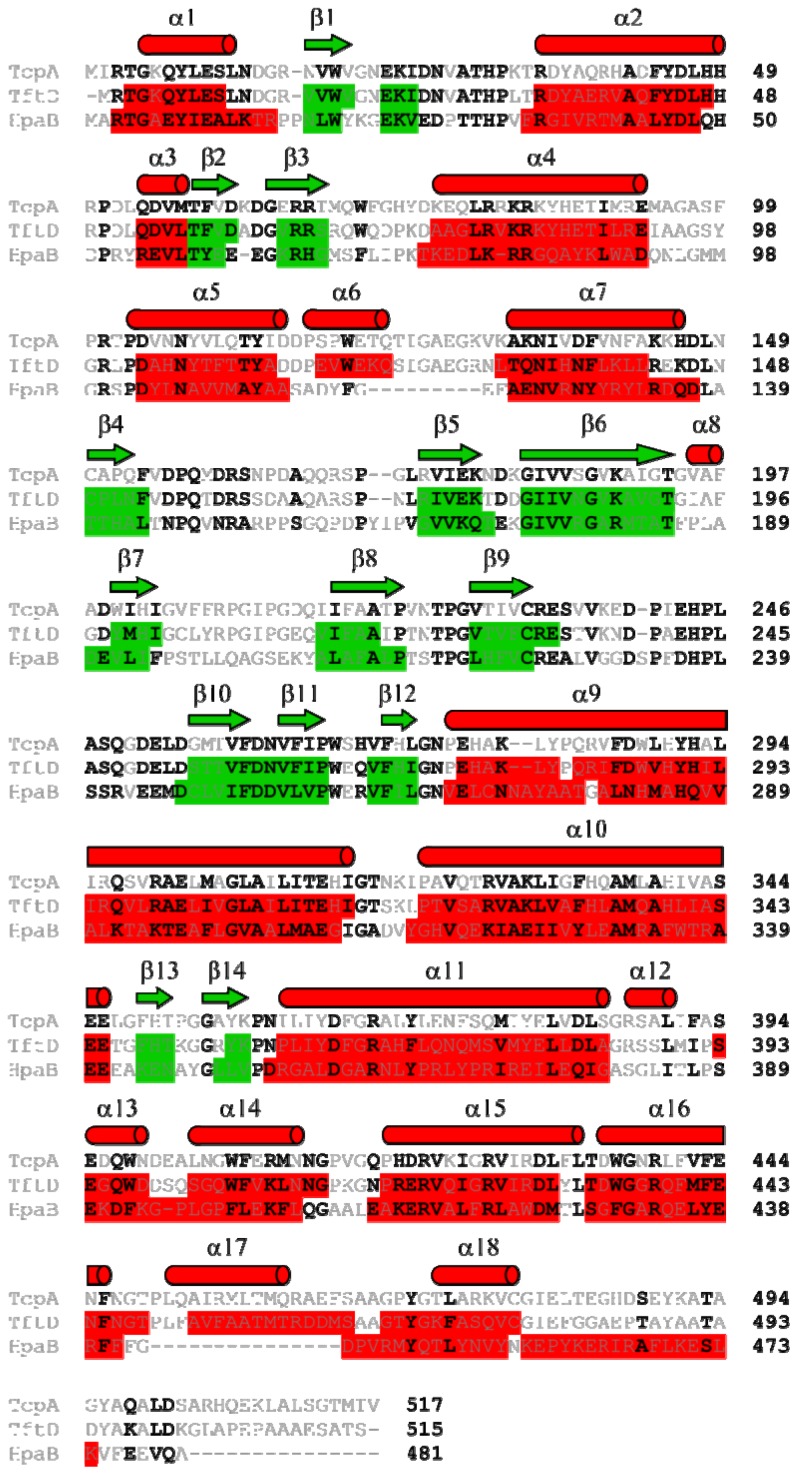
Amino acid sequence and secondary structure alignment of TcpA with other flavin-dependent monooxygenases. Secondary structure elements for TcpA are depicted as red cylinders and green arrows for α-helices and β-strands, respectively. Secondary structural elements for TftD and HpaB are also highlighted in colors (red for α-helices and green for β-strands). Conserved residues among those monooxygenases are shown in boldface. TcpA, 2,4,6-trichlorophenol monooxygenase from *Cupriavidus necator* JMP134; TftD, chlorophenol 4-monooxygenase; HpaB, 4-hydroxyphenylacetate 3-monooxygenase from *T. thermophilus* HB8.

The position of the riboflavin moiety of the FADH_2_ was readily obtained through the combination of superposition with the 4-hydroxylphenylacetate 3-monooxygenase (HpaB) from *Thermus thermophilus* HB8 (2YYL) [32]. TcpA and HpaB share 25% sequence identity and conservation of nearly all secondary structural elements with a Cα alignment RMSD of 1.55 Å (Figure 5). These coordinates were used as an initial placement for submission to Autodock 4.0 as described in the methods section. The docked position of the FADH_2_ riboflavin moiety was ideal in both chemical and stereo fit, as illustrated by molecular surfacing (Figure 4 inset). The hydroxyl group of Thr193, which is conserved in TftD and HpaB, was within a hydrogen bond distance of 3.1 Å from the N5 atom of the isoalloxizine ring. The guanidinium side chain of Arg101 was 4.5 Å from the C4α atom of the isoalloxizine ring of FADH_2_. We previously proposed that FADH_2_ is immediately oxidized to hydroperoxyflavin intermediate, FADHOOH [27], which is then stabilized by the enzyme. Our docking result confirmed the ability for hydrogen bonding between R101 and the hydroperoxyflavin which could ultimately stabilize the intermediate as seen with TftD and HpaB [32,33]. The transiently stable hydroperoxyflavin was located at the proper position for immediate reaction with bound TCP. FADH_2_ created the binding pocket for the incoming substrate together with TcpA, which was consistent with the previous data that there is no affinity for the substrate without a preexisting FADH_2_ molecule.

### 2.3. Substrate Binding

**Figure 6:**
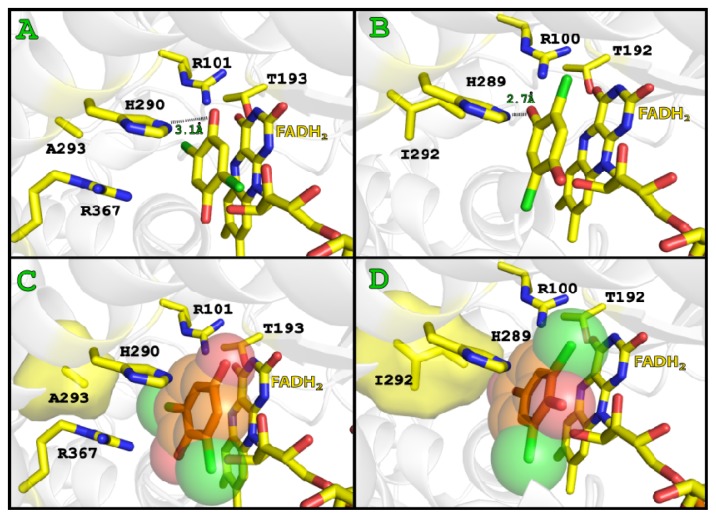
(**a**) TcpA with docked FADH_2_ and 2,5-DiCHQ (**b**) TftD with docked FADH_2_ and 2,5-DiCHQ (**c**) TcpA with docked FADH_2_ and 2,6-DiCHQ (**d**) TftD with FADH_2_ and docked 2,6-DiCHQ.

Through Autodock 4.0, various substrates such as 2,4,5-TCP, 2,5-DiCHQ, 2,4,6-TCP and 2,6-DiCBQ were docked into both TcpA and TftD with FADH_2_ prepositioned as described above. The protein and FADH_2_ remained as rigid receptors while the above listed substrates were treated as flexible ligands. Several key residues were identified that helped to explain the differences in both mechanism and substrate specificity between TcpA and TftD. The lowest energy conformations of 2,4,5-TCP and 2,5-DiCHQ docked to both TftD and TcpA illustrated the *para* position chlorine or hydroxyl group, respectively, were located within hydrogen bonding distance of His289/His290 (Figure 6a,b), the previously determined catalytic residue [33]. TcpA docking with 2,4,6-TCP and 2,6-DiCHQ also illustrated the *para* position chlorine or hydroxyl group, respectively, oriented within hydrogen bonding distance of His290 as expected (Figure 6c). One critical point was noted regarding the TftD-docking result with both 2,6-DiCHQ and 2,4,6-TCP as the substrates. The position of 2,4,6-TCP in the active site of TftD allowed the *para* position chlorine group to be within hydrogen bonding distance of His289. This would allow for productive dechlorination at the 4-position as suggested previously [32]. The angular position of 2,6-DiCHQ was also very similar to 2,4,6-TCP, however, in order for productive dechlorination to occur, the orientation would have to be reversed to allow His289 to act as a general base and abstract a proton from the 1-position hydroxyl group. This 1-position hydroxyl moiety of 2,6-DiCHQ was seen facing out of the pocket in an unproductive position for further catalysis (Figure 5d).

### 2.4. Substrate Binding Site and Reaction Mechanism: Differences between TftD and TcpA

**Figure 7:**
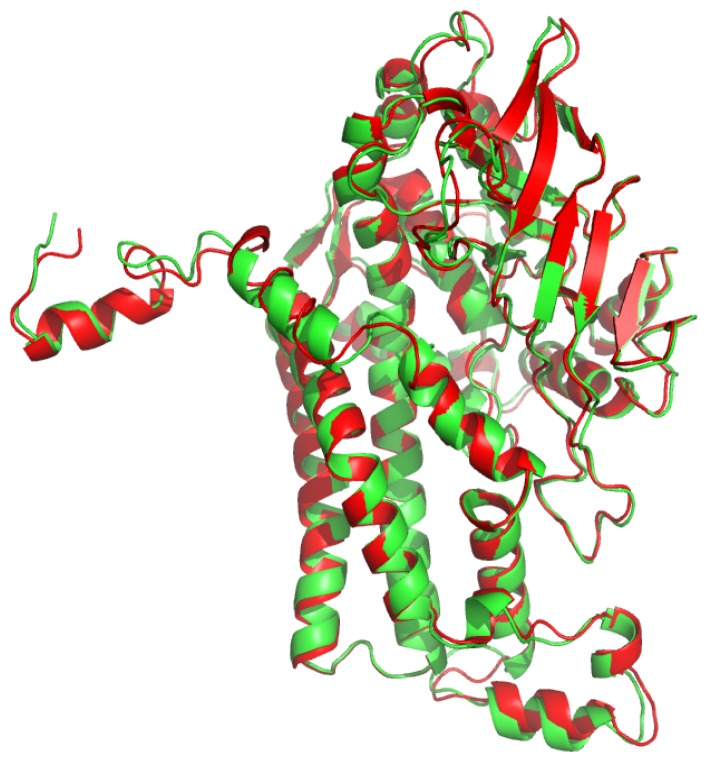
Ribbon diagram structural alignment of TcpA and TftD monomers. The C-α backbone atoms of TcpA (**green**) and TftD (**red**) (PDB: 3HWC) were aligned illustrating high similarity in secondary structural elements. This alignment was performed using Open-Source PyMOL™ (version 1.3).

A structural alignment of both TcpA and TftD showed that the global structure of the two enzymes were quite similar with an r.m.s.d. of 0.78 between Cα atoms (Figure 7). Thus it is very likely that the apparent differences in their catalytic activity and substrate specificity stem from several proposed amino acid residues indentified by our docking studies. TcpA first catalyzes a monooxygenase reaction to remove the chlorine at the 4-position and then removes the chlorine at the 2-position by a hydrolytic reaction. On the other hand, TftD can only perform the oxidative dechlorination despite their high similarity in amino acid sequence.

The docking results of TftD with 2,4,5-TCP, 2,4,6-TCP, 2,5-DiCHQ and 2,6-DiCHQ all had similar angular orientations as expected along the planar axis of the ring. The lowest energy cluster of all above-mentioned substrates showed the hydroxyl group at the 4-position of each ring within hydrogen bonding distance of His289. This supports the previous result of site directed mutagenesis [33] suggesting that His289 can act as a general base and abstract a proton from the 4-position hydroxyl group in 2,5-DiCHQ or the hydroxyl group introduced to the 4-position by the flavoperoxy intermediate in 2,4,5-TCP and 2,4,6-TCP. One critical residue difference noted underneath the docked 2,6-DiCHQ was Ile292 in TftD and Ala292 in TcpA (Figure 6). 2,6-DiCHQ in TcpA had the 2- or 6-position chlorine atom in a pocket created by alanine while still allowing the 4-position hydroxyl group to be in hydrogen bonding with H290. 2,6-DiCHQ docked to TftD had a different orientation such that the 2- or 6-position chlorine atom could be accommodated, however the 1-position hydroxyl group could not be reached by His289. It appeared that Ile292 in TftD causes steric hindrance with the 2- or 6-position chlorine atom ultimately preventing coordination of the 1-position hydroxyl group with His289 and thus further catalytic dechlorination. It is likely that 2,6-DiCBQ and 2,5-DiCBQ are released from the binding pocket of TftD due to the less polar character of the carbonyl group in the produced quinone. Both 2,6-DiCBQ and 2,5-DiCBQ would be reduced into the corresponding quinol form probably outside of the enzyme active site and reentered/coordinated by His289. However, only 2,5-DiCHQ can properly orient itself in the second dechlorination position due to the above-mentioned steric hindrance generated by Ile292 in TftD.

**Figure 8:**
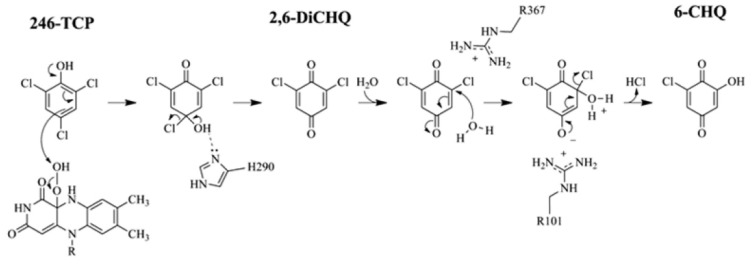
Proposed mechanism of TcpA.

With a same logic as above, TcpA can hold 2,6-DiCBQ longer, which allows it to be further hydrolyzed to 6-chloro-2-hydroxy-*p*-benzoquinone. The 2- or 6-Cl atom in the quinone form, which contains α,β-unsaturated carbonyl moiety, is much more susceptible to displacement by a nucleophile than the same chlorine atoms of 2,4,6-TCP. The angular orientations of 2,4,6-TCP and 2,6-DiCHQ in TcpA were relatively similar ultimately suggesting that perhaps the substrate does not have to reorient following the primary dechlorination. The docked structure of 2,6-DiCHQ illustrated His290 and Arg101 within hydrogen bonding distance of the *para*-hydroxyl group. The electrophilicity of the corresponding α,β-unsaturated carbonyl moiety at the 2-chlorine position should be slightly elevated through inductive effects caused by the properly oriented guanidinium group of Arg367. Due to the more hydrophobic nature of the TcpA binding pocket, the product of the first reaction in quinone form can stay longer in TcpA than in TftD allowing sufficient time to be attacked by a water molecule. In addition, the hydrogen bond between Arg101 and the *para*-hydroxyl group would lead to charge stabilization during the attack of a water molecule at the 2- or 6-position chorine (Figure 8). Ultimately this would lead to hydrolytic dechlorination immediately following an oxygenase dechlorination without reorientation of the substrate or a need for the substrate to leave the active site.

## 3. Experimental Section

### 3.1. Expression and Purification

TcpA was cloned into the overexpression plasmid pET30 LIC (Novagen) as previously described [12]. Expression of TcpA was carried out by inoculating 100 mL of LB supplemented with 30 μg/mL kanamycin from a freezer stock of pET30TcpA in BL21 (DE3) cells. This was allowed to grow overnight at 37 °C with constant shaking, after which this culture was used to inoculate 1.5 liters of LB medium. Protein expression was induced by addition of isopropyl β-D-thiogalactopyranoside to 0.5 mM final concentration at mid-log phase (A_600_ = ~0.6). Following induction the cell line was incubated for 12 h at 22 °C with constant shaking at 250 rpm. The cells were then harvested by centrifugation (3,000× *g*), after which the pellet was frozen to promote cell lysis. The pellet was thawed at room temperature and resuspended in a minimal volume of lysis buffer (50 mm Tris (pH 8.0), 300 mm NaCl, 20 mm imidazole and 1mM dithiothreitol (DTT)), sonicated 5 times for 10 seconds each using a model 450 Sonifier^®^ (Branson Ultrasonics), and the resulting lysate cleared by centrifugation (20,000× *g* for 45 min).

Lysate was then applied to a nickel-nitrilotriacetate column and washed with several column volumes of lysis buffer. Elution took place with the lysis buffer containing 250 mM imidazole. Eluted fractions containing TcpA were combined, concentrated, and buffer-exchanged into 20 mM Tris (pH 8.5) with 1mM DTT by ultrafiltration in an Amicon 8050 cell with a 10-kDa cutoff membrane (Millipore) and loaded onto a Mono Q™ GL10/100 anion-exchange column (GE Healthcare), and eluted with a linear NaCl gradient at ~150 mm NaCl. Fractions containing TcpA were pooled, concentrated, and exchanged into 20 mM Tris (pH 8.5) with 1mM DTT.

With the exception of the nickel-nitrilotriacetate column, all purification steps were carried out using an Amersham Biosciences/BioCad 700E preparative HPLC (Applied Biosystems). All purification steps were monitored and final homogeneity (>99%) was estimated using SDS-PAGE and Coomassie blue staining.

### 3.2. Crystallization and Data Collection

For crystallization, the buffer of the purified TcpA solution was replaced with a solution containing 20 mM Tris (pH 8.0) with 1 mM DTT, and the protein concentration was adjusted to 10 mg mL^−1^. Crystals of TcpA were grown at 4 °C using the hanging drop vapor diffusion method. 1.5 μL of TcpA was mixed with 1.5 μL of reservoir solution (20% (w/v) polyethylene glycol 3350 (pH 7.4) with 0.2 M potassium sodium tartrate tetrahydrate) and equilibrated against the reservoir. Diffraction quality crystals appeared after two months. The TcpA crystal belongs to the monoclinic space group C2 with four molecules in an asymmetric unit. The native data of 2.0 Å resolution was collected from the Advanced Light Source at a temperature of −170 °C (ALS, beam line 8.2.1).

### 3.3. Structure Determination and Refinement

The structure of TcpA was solved by molecular replacement using the coordinates of the TftD [33] and the software package AMORE [34]. The rigid-body refinement of the initial position was carried out using 15.0 Å to 3.0 Å resolution data and produced an R-value of 29%. After several cycles of positional refinement, temperature factor refinement, and simulated annealing omit map, most of the residues were able to be fitted to the electron density. The tracing for loop areas and the density fitting for the remaining side chain were performed using series of improved 2F_o_-F_c_ and omit maps utilizing the software Coot [35]. The residues 156–170 are disordered and the corresponding electron density was not visible from the early stage of refinement. The model was then refined with 2.5 Å resolution data using Phenix [36]. The coordinates and diffraction data of the TcpA and have been deposited in the Protein Data Bank (4G5E).

### 3.4. Molecular Docking of FADH_2_ and Substrates

The molecular docking process was performed with Autodock 4.0 [37]. Python Molecule Viewer (PMV) suite was used to convert the structure files of FADH_2_ and the substrates to PDBQT format. The three dimensional structures of FADH_2_ and all substrates were extracted from the Pubchem compound web server; 2,5-DiCHQ:CID65, 2,4,5-TCP:CID7271, 2,6-DiCHQ:CID88366, 2,4,6-TCP:CID6914 and FAD:CID643975. Partial charges were added through PMV and the default number of torsions was selected for searching the conformational space. The docking was performed in a rigid receptor–flexible ligand model, where full flexibility was given to the small molecule while the whole protein and aligned FADH_2_ were kept rigid. Individual atomic affinity grids were calculated for each substrate atom type *versus* every atom type present in the protein and FADH_2_ with uniform spacing of 0.375 Å between each grid point. At least 100–250 docked conformations were generated in different grid sizes using Lamarckian genetic algorithm search method employed in Autodock. All other conformation search parameters were kept default. The rigid receptor TcpA or TftD with docked FADH_2_ was used for substrate docking studies.

### 3.5. Molecular Mass Determination

The weight-average molecular mass of TcpA was measured by combined size exclusion chromatography and multi-angle laser light scattering as described previously [38]. Briefly, 100 μg of TcpA was loaded onto a BioSep-SEC-S 2000 column (Phenomenex) and eluted isocratically with a flow rate of 0.5 mL min^−1^. The eluate was passed through tandem UV detector (Gilson), Optilab DSP interferometric refractometer (Wyatt Technology), and a Dawn EOS laser light scattering detector (Wyatt Technology). Scattering data was analyzed using the Zimm fitting method with software (ASTRA) provided by the instrument manufacturer.

## 4. Conclusions

2,4,6-Trichlorophenol-4-monoxygenase is a unique enzyme that catalyzes the sequential dechlorination of 2,4,6-TCP to 6,-CHQ in a single active site through both oxydative and hydrolytic reactions. All or part of the residues discussed above determine the orientation and/or position of 2,4,6-TCP and 2,4,5-TCP, which, in turn, could affect the resulting product of the dechlorination reaction. The high global structural similarity between TcpA and TftD suggests the catalytic variation must stem from differences in the active site. Our current work includes complex crystallizations of TcpA and TftD with substrate analogues and FADH_2_ to confirm the angular orientations of the substrate molecules, which will further guide site-directed mutagenesis studies. Therefore this comprehensive knowledge will definitely provide insight into microbial dechlorination mechanisms and could be used to further broaden the catalytic capability of those enzymes.

## Figures and Tables

**Table 1:** Summary of crystallographic data.

Data	Apo
Wavelength (Å)	1.00
Resolution (Å)	49.5–2.0
Space group	C2
Cell dimensions (Å)	*a* = 107.07 *b* = 180.05 *c* = 110.20 α = 90.00 β = 97.87 γ = 90.00
Asymmetric unit	4
Total observations	437,758
Unique reflections	126,447
Completeness (%)	97.4 (75.5)
R_sym_ a,b	0.010 (0.898)
Refinement	
Resolution (Å)	49.5–2.5
Number of reflections	67,560 (95%, >1σI)
R_cryst_ c	17.4
R_free_ d	22.4
r.m.s.d. bonds (Å)	0.008
r.m.s.d. angles (º)	1.081
Ramachandran Plot (%)	
Preferred	95.18
Allowed	3.91
Outliers	0.91
Number of atoms	
Protein	15,103
Water	315

a Numbers in parentheses refer to the highest resolution shell;

b R_sym_ = ∑*I*_h_ − <*I*_h_>|/∑*I*_h_, where <*I*_h_> is the average intensity over symmetry equivalent reflections;

c R_cryst_ = ∑|F_obs_ − F_calc_|/∑F_obs_, where summation is over the data used for refinement;

d R_free_ was calculated as for R_cryst_ using 5% of the data that was excluded from refinement.

## References

[1] Czaplicka M. (2004). Sources and transformations of chlorophenols in the natural environment. Sci. Total Environ.

[2] U.S. EPA Integrated Risk Information System (IRIS) on 2,4,6-Trichlorophenol. National Center for Environmental Assessment, Office of Research and Development.

[3] Xun L., Wagon K. (1995). Purification and properties of 2,4,5-trichlorophenoxyacetate oxygenase from Pseudomonas cepacia AC1100. Appl. Environ. Microbiol.

[4] Bock C., Kroppenstedt R.M., Schmidt U., Diekmann H. (1996). Degradation of prochloraz and 2,4,6-trichlorophenol by environmental bacterial strains. Appl. Microbiol. Biotech.

[5] Crosby D.G. (1981). Environmental chemistry of pentachlorophenol. Pure Appl. Chem.

[6] Firestone D. (1978). The 2,3,7,8-trtrachlorodibenzo-para-dioxin problem: A review. Stockh. Ecol. Bull.

[7] Hoekstra E., de Weerd H., de Leer E., Brinkman U. (1999). Natural formation of chlorinated phenols, dibenzo-*p*-dioxins, and dibenzofurans in soil of a Douglas fir forest. Environ. Sci. Technol.

[8] Kintz P., Tracqui A., Mangin P. (1992). Accidental death caused by the absorption of 2,4-dichlorophenol through the skin. Arch. Toxicol.

[9] Agency for Toxic Substances and Disease Registry (ATSDR) (1999). Toxicological Profile for Chlorophenols.

[10] Kilbane J., Chatterjee D., Chakrabarty A. (1982). Biodegradation of 2,4,5-trichlorophenoxyacetic acid by a pure culture of Pseudomonas cepacia. Appl. Environ. Microbiol.

[11] Kellogg S., Chatterjee D., Chakrabarty A. (1981). Plasmid-assisted molecular breeding: New technique for enhanced biodegradation of persistent toxic chemicals. Science.

[12] Louie T., Webster C., Xun L. (2002). Genetic and biochemical characterization of a 2,4,6-trichlorophenol degradation pathway in Ralstonia eutropha JMP134. J. Bacteriol.

[13] Xun L., Webster C.M. (2004). A monooxygenase catalyzes sequential dechlorinations of 2,4,6-trichlorophenol by oxidative and hydrolytic reactions. J. Biol. Chem.

[14] Xun L. (1996). Purification and characterization of chlorophenol 4-monooxygenase from Burkholderia cepacia AC1100. J. Bacteriol.

[15] Reineke W., Knackmuss J.-J. (1988). Microbial degradation of haloaromatics. Ann. Rev. Microbiol.

[16] Husain M., Entsch B., Ballou D.P., Massey V., Chapman P.J. (1980). Fluoride elimination from substrates in hydroxylation reaction catalyzed by p-hydroxybenzoate hydroxylase. J. Biol. Chem.

[17] Dai M., Rogers J.B., Warner J.R., Copley S.D. (2003). A previously unrecognized step in pentachlorophenol degradation in Sphingobium chlorophenolicum is catalyzed by tetrachlorobenzoquinone reductase (PcpD). J. Bacteriol.

[18] Haigler B.E., Suen W.C., Spain J.C. (1996). Purification and sequence analysis of 4-methyl-5-nitrocatechol oxygenase from Burkholderia sp. strain DNT. J. Bacteriol.

[19] Liu Y., Louie T., Xun L. (2001). Purification and characterizatoin of iminodiacetate-dehydrogenase from the EDTA-degrading strain BNC1. Appl. Environ. Microbiol.

[20] Perry L., Zylstra G. (2007). Cloning of a gene cluster involved in the catabolism of p-nitrophenol by Arthrobacter sp. strain JS443 and characterization of the p-nitrophenol monooxygenase. J. Bacteriol.

[21] Otto K., Hofstetter K., Röthlisberger M., Witholt B., Schmid A. (2004). Biochemical characterization of StyAB from *Pseudomonas* sp. strain VLB120 as a two-component flavin-diffusible monooxygenase. J. Bacteriol.

[22] Galán B., Díaz E., Prieto M., García J. (2000). Functional analysis of the small component of the 4-hydroxyphenylacetate 3-monooxygenase of Escherichia coli W: A prototype of a new Flavin:NAD(P)H reductase subfamily. J. Bacteriol.

[23] Kirchner U., Westphal A., Müller R., van Berkel W. (2003). Phenol hydroxylase from Bacillus thermoglucosidasius A7, a two-protein component monooxygenase with a dual role for FAD. J. Biol. Chem.

[24] Malito E., Alfieri A., Fraaije M., Mattevi A. (2004). Crystal structure of a Baeyer-Villiger monooxygenase. Proc. Natl. Acad. Sci. USA.

[25] Li L., Liu X., Yang W., Xu F., Wang W., Feng L., Bartlam M., Wang L., Rao Z. (2008). Crystal structure of long-chain alkane monooxygenase (LadA) in complex with coenzyme FMN: Unveiling the long-chain alkane hydroxylase. J. Mol. Biol.

[26] Valton J., Fontecave M., Douki T., Kendrew S., Nivière V. (2006). An aromatic hydroxylation reaction catalyzed by a two-component FMN-dependent Monooxygenase. The ActVA-ActVB system from Streptomyces coelicolor. J. Biol. Chem.

[27] Gisi M., Xun L. (2003). Characterization of chlorophenol 4-monooxygenase (TftD) and NADH: Flavin adenine dinucleotide oxidoreductase (TftC) of Burkholderia cepacia AC1100. J. Bacteriol.

[28] Massey V. (1994). Activation of molecular oxygen by flavins and flavoproteins. J. Biol. Chem.

[29] Filisetti L., Fontecave M., Nivière V. (2003). Mechanism and substrate specificity of the flavin reductase ActVB from Streptomyces coelicolor. J. Biol. Chem.

[30] Gao B., Ellis H. (2005). Altered mechanism of the alkanesulfonate FMN reductase with the monooxygenase enzyme. Biochim. Biophys. Res. Commun.

[31] Nissen M., Youn B., Knowles B., Ballinger J., Jun S., Belchik S., Xun L., Kang C. (2008). Crystal structures of NADH:FMN oxidoreductase (EmoB) at different stages of catalysis. J. Biol. Chem.

[32] Kim S., Hisano T., Takeda K., Iwasaki W., Ebihara A., Miki K. (2007). Crystal structure of the oxygenase component (HpaB) of the 4-hydroxyphenylacetate 3-monooxygenase from Thermus thermophilus HB8. J. Biol. Chem.

[33] Webb B.N., Ballinger J.W., Kim E., Belchik S.M., Lam K.-S., Youn B., Nissen M.S., Xun L., Kang C. (2010). Characterization of Chlorophenol 4-Monooxygenase (TftD) and NADH:FAD Oxidoreductase (TftC) of Burkholderia cepacia AC1100. J. Biol. Chem.

[34] Navaza J. (2001). Implementation of molecular replacement in AMoRe. Acta Crystallogr. Sect.

[35] Emsley P., Cowtan K. (2004). Coot: Model-Building tools for molecular graphics. Acta Crystallogr. Sect.

[36] Brunger A.T., Adams P.D., Clore G.M., DeLano W.L., Gros P., Grosse-Kunstleve R.W., Jiang J.-S., Kuszewski J., Nilges M., Pannu N.S. (1998). Crystallography & NMR System: A new software suite for macromolecular structure determination. Acta Crystallogr. Sect.

[37] Sanner M.F. (1999). Python: A programming language for software integration and development. J. Mol. Graph. Model.

[38] Youn B., Moinuddin S., Davin L., Lewis N., Kang C. (2005). Crystal structures of apo-form and binary/ternary complexes of Podophyllum secoisolariciresinol dehydrogenase, an enzyme involved in formation of health-protecting and plant defense lignans. J. Biol. Chem.

